# Public health round-up

**DOI:** 10.2471/BLT.17.011017

**Published:** 2017-10-01

**Caribbean communities devastated by tropical storms**

A boy rides a bicycle through the town of Ouanaminthe in Haiti, where thousands of homes and large swathes of land were covered with mud by Hurricane Irma as it swept across the eastern Caribbean last month leaving a trail of destruction.

**Figure:**
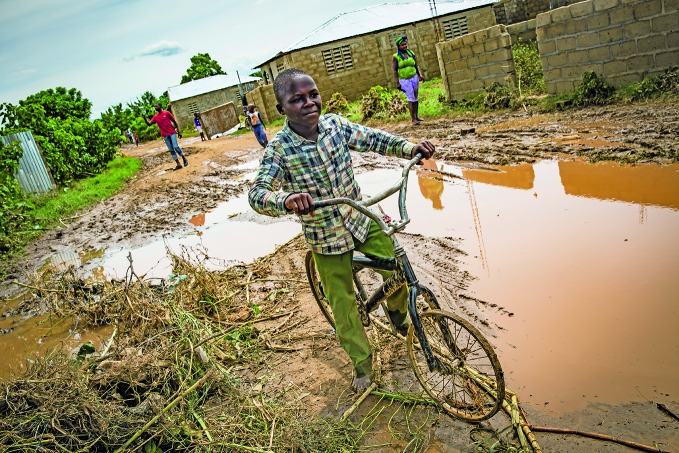
© UNICEF/UN0119394/M. Bradley

## Make mercury history

Officials from more than 140 countries met last month to consider how to implement a new global treaty to reduce the harmful effects of mercury for human health and the environment.

The Minamata Convention on Mercury came into force on 18 August and the first meeting of the Conference of the Parties to the convention was convened by United Nations Environment in Geneva from 24 to 29 September under the banner “make mercury history”.

The World Health Organization (WHO) is tasked with providing advice and technical support to its Member States to help them make the treaty a reality, according to a 2014 World Health Assembly resolution (WHA 67.11).

By 2020, parties to the convention are expected to draw up plans to reduce and, if possible, eliminate the use of mercury in small-scale gold mining operations and set thresholds for emissions of mercury from industrial sources, including coal fired power plants.

They should also reduce the use of dental amalgam, which contains mercury, and phase out the manufacture, import and export of skin lightening cosmetics, thermometers and blood pressure measuring devices containing mercury.

An important exception is thiomersal (ethyl-mercury), used in some human and animal vaccines, as there is no evidence that the amount of thiomersal used in vaccines poses a health risk.

Mercury is a heavy metal that travels long distances in the atmosphere, persists in the environment and accumulates in ecosystems, including in fish. Mercury exposure in humans can have serious health consequences, including permanent damage to the developing nervous system in children.

www.who.int/mediacentre/events/2017/make-mercury-history/en/

## Investing in the early years

A new WHO and UNICEF initiative aims to promote early childhood development, by encouraging and supporting parents and caregivers to provide nurturing care for children during the first months and years of their lives. 

Nurturing care refers to interventions that meet children's health and nutritional needs, and that provide opportunities for early learning and interactions that are emotionally supportive and developmentally stimulating. 

Studies show that investing in such care, especially during the first three years of a child’s life, starting from conception, can have lasting benefits for health, productivity and social cohesion. 

The initiative was presented at a side-event at the United Nations (UN) General Assembly in New York last month. 

It was the first in a series of consultations to introduce the idea of a WHO–UNICEF nurturing care framework for action and results and to seek feedback on this from governments, civil society, health and child development professionals and other stakeholders. 

The final document will be launched in May 2018, and discussed as part of an agenda item on the *Global strategy for women’s, children’s and adolescents’ health* at the WHO’s Executive Board and the World Health Assembly next year. 

www.who.int/nurturingcarechild

## Preventing cholera 

A preventive cholera vaccination campaign targeting half a million people living in areas affected by recent floods and landslides was planned to start in Freetown, Sierra Leone last month.

In the country’s last major cholera outbreak in 2012, 392 people died and more than 25 000 were infected. Although cholera had not been reported in Sierra Leone as of 11 September, recent landslides and floods have disrupted the infrastructure and substantially increased the risk of a cholera outbreak.

A preventive vaccination campaign, with two rounds of oral cholera vaccine was planned by the Government of Sierra Leone, with support from the Gavi Alliance, WHO, UNICEF and other partners. The first vaccination round was due to take place in September and the second round, early this month.

A decision to release cholera vaccine from the global stockpile for the preventive campaign was taken on 31 August, by the International Coordinating Group for Vaccine Provision, following a WHO assessment of the situation. Just over one million doses of the vaccine were delivered to Freetown on 7 September by UNICEF.

WHO recommends that vaccination against cholera should be considered in emergencies and other situations, where there is a high risk of disease outbreaks and when vaccination can be combined with standard prevention and control measures for the disease.

These measures include readiness to provide testing and treatment, steps to ensure access to safe water and sanitation, and community mobilization to engage the public in efforts to prevent infection.

Since the global stockpile of cholera vaccine was established in 2013, more than 14 million doses have been released for deployment in 17 countries.

www.who.int/mediacentre/news/releases/2017/sierra-leone-cholera-vaccination

## WHO report tracks NCDs

Governments need to step up their efforts to control noncommunicable diseases (NCDs) to meet global targets set out in the Agenda for Sustainable Development, according to the *Noncommunicable diseases progress monitor 2017*.

Progress has been made in setting national NCD targets, but many countries need to make better use of so-called best-buy approaches – the cost-effective interventions for reducing NCDs that were endorsed by the World Health Assembly this year, the report found.

The report, which was released last month, is the latest in a WHO series charting progress countries have made in addressing the epidemics of cardiovascular and chronic respiratory diseases, cancer and diabetes since 2011. It provides data on 10 indicators in all of WHO’s 194 Member States, based on the results of a recent survey.

NCDs represent the largest cause of death globally and kill 17 million people before the age of 70 annually. Many NCD deaths can be prevented by reducing tobacco use, unhealthy diets, physical inactivity and harmful use of alcohol.

The findings of the *Noncommunicable diseases progress monitor 2017* will underpin a report that WHO is due to submit to the UN Secretary General António Guterres later this year, ahead of the third UN High-level Meeting on NCDs in 2018.

www.who.int/nmh/

**Cover photo**

These girls fled the village of Habile where they lived in Chad after it was attacked in December 2006. This photograph shows them returning to Habile to fetch some remaining belongings and fire wood.

**Figure:**
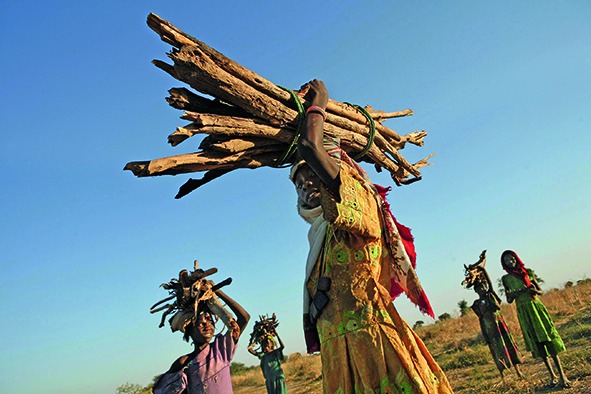
©UNHCR/Hélène Caux

## Media can help prevent suicide

Media professionals can play an important role in the prevention of suicide, according to WHO guidance updated to emphasize the positive effect media professionals can play when reporting suicides.

Journalists can help prevent suicidal behaviour by providing information about where to seek help, providing the facts on suicide and suicide prevention, reporting stories of how people were able to cope with suicidal thoughts and how they can get help.

According to the WHO guidance, the media should not place news stories about suicide prominently or unduly repeat such stories, nor should they sensationalize suicide or present suicide as a solution to people’s problems.

“The scientific evidence is clear: irresponsible news reporting of suicides can increase the number of suicides and suicide attempts,” said Alexandra Fleischmann, from WHO’s Department of Mental Health and Substance Abuse. “We hope that journalists will consult the WHO guidance *Preventing suicide: a resource for media professionals*, as the media can play a crucial role in suicide prevention.”

Journalists should avoid describing suicide methods in detail and revealing the location where suicides occurred and should not provide photographs, video footage or social media links of such incidents as vulnerable persons may imitate the act.

www.who.int/mental_health/suicide-prevention/resource_booklet_2017

## Fewer smokers in the Russian Federation and Ukraine

Fewer people are smoking in the Russian Federation and Ukraine thanks to tobacco-control measures in line with the WHO Framework Convention on Tobacco Control (WHO FCTC).

According to the results of the Global Adult Tobacco Survey, conducted in the Russian Federation in 2009 and again in 2016, tobacco use decreased among adults from 39.4% in 2009 to 30.9% in 2016 (from 60.7% to 50.9% among males and from 21.7% to 14.3% among females). 

The Russian Federation, a country of 143 million people, introduced tobacco-control measures in 2013 and 2014. 

Since then second-hand smoke in homes and public places has declined, exposure to tobacco advertising, promotion and sponsorship has decreased and the average price of a packet of 20 cigarettes has increased more than three fold.

The Global Adult Tobacco Survey results in Ukraine showed equally dramatic results. In 2017, an estimated 7.2 million adult Ukrainians smoke daily (35.9% of adult men and 7.0% of adult women) as compared with 10.5 million adults who smoked daily in 2010 (45.4% of adult men and 8.9% of adult women).

The proportion of the population exposed to second-hand smoke at home, at work and in public, and of those exposed to tobacco promotion through marketing of tobacco products, have both decreased. Tobacco product prices have increased significantly, partly because of tax increases, and the public is more aware of the harms of tobacco, the survey found.

www.euro.who.int/en/health-topics/disease-prevention/tobacco/data-and-statistics

**Looking ahead**

18–20 October – WHO Global Conference on Noncommunicable Diseases. Montevideo, Uruguay

1–3 November – World Hepatitis Summit 2017. São Paulo, Brazil

16–17 November – Global Ministerial Conference on Tuberculosis. Moscow, Russian Federation

